# *Alu* Sequences in Undifferentiated Human Embryonic Stem Cells Display High Levels of A-to-I RNA Editing

**DOI:** 10.1371/journal.pone.0011173

**Published:** 2010-06-21

**Authors:** Sivan Osenberg, Nurit Paz Yaacov, Michal Safran, Sharon Moshkovitz, Ronit Shtrichman, Ofra Sherf, Jasmine Jacob-Hirsch, Gilmor Keshet, Ninette Amariglio, Joseph Itskovitz-Eldor, Gideon Rechavi

**Affiliations:** 1 Cancer Research Center, Chaim Sheba Medical Center, Tel Hashomer, Israel; 2 Sackler School of Medicine, Tel Aviv University, Tel Aviv, Israel; 3 Sohnis and Forman Families Stem Cell Center, Bruce Rappaport Faculty of Medicine, Technion-Israel Institute of Technology, Haifa, Israel; 4 Department of Obstetrics and Gynecology, Rambam Medical Center, Haifa, Israel; University of Poitiers, France

## Abstract

Adenosine to Inosine (A-to-I) RNA editing is a site-specific modification of RNA transcripts, catalyzed by members of the ADAR (Adenosine Deaminase Acting on RNA) protein family. RNA editing occurs in human RNA in thousands of different sites. Some of the sites are located in protein-coding regions but the majority is found in non-coding regions, such as 3′UTRs, 5′UTRs and introns - mainly in *Alu* elements. While editing is found in all tissues, the highest levels of editing are found in the brain. It was shown that editing levels within protein-coding regions are increased during embryogenesis and after birth and that RNA editing is crucial for organism viability as well as for normal development. In this study we characterized the A-to-I RNA editing phenomenon during neuronal and spontaneous differentiation of human embryonic stem cells (hESCs). We identified high editing levels of *Alu* repetitive elements in hESCs and demonstrated a global decrease in editing levels of non-coding *Alu* sites when hESCs are differentiating, particularly into the neural lineage. Using RNA interference, we showed that the elevated editing levels of *Alu* elements in undifferentiated hESCs are highly dependent on ADAR1. DNA microarray analysis showed that ADAR1 knockdown has a global effect on gene expression in hESCs and leads to a significant increase in RNA expression levels of genes involved in differentiation and development processes, including neurogenesis. Taken together, we speculate that A-to-I editing of *Alu* sequences plays a role in the regulation of hESC early differentiation decisions.

## Introduction

Human embryonic stem cells (hESCs) are derived from the inner cell mass of blastocysts [1], [2] Their ability to grow for long periods, while preserving normal karyotype and pluripotency holds enormous potential for these cells to become important tools in cell differentiation and early developmental research, drug discovery and for future regenerative medicine. The pluripotency of these cells can be easily demonstrated when they are grown in suspension where they spontaneously differentiate and form aggregates named Embryoid Bodies (EBs) in modes which recapitulate early events of embryonic development [3], [4]. It has been shown that mature EBs include many types of cells which represent derivatives of the three embryonic germ layers [3], [4]. In addition, it was shown that by manipulating their growth conditions in specific ways, ESCs differentiation can be directed toward specific lineages by similar mechanisms to those occurring *in vivo* [5].

The transcriptome and the proteome diversity have been shown to be regulated by post-transcriptional RNA processing mechanisms; the best studied being alternative splicing [6]. RNA editing is another post transcriptional processing mechanism. It generates RNA sequences that are different from the ones encoded by the genome, and thereby contributes to the diversity of gene products [7], [8]. It was shown by our group as well as by other researchers that RNA editing is a global phenomenon, affecting thousands of genes [9], [10], [11], [12], [13]. RNA editing increases significantly the complexity of transcription products and has a major influence on cell physiology [7], [8]. The most common editing type is the conversion of Adenosine to Inosine (A–to-I) by hydrolytic deamination in double strand RNA regions [7], [9], [10], [11], [12]. A–to–I RNA editing is processed by enzymes that belong to the ADAR (Adenosine Deaminase Acting on RNA) protein family and are encoded by the ADAR1, ADAR2 and ADAR3 genes [7]. ADAR1 and ADAR2 are expressed ubiquitously and have an active deaminase domain. In contrast, ADAR3 is expressed only in the brain and its activity as an editing enzyme remains to be demonstrated [7]. ADAR1 has two protein isoforms. ADAR1 p110 is located exclusively in the nucleus and its RNA is transcribed from the constitutive promoters 1B and 1C. In contrast, ADAR1 p150 is located both in the cytoplasm and in the nucleus and its RNA is transcribed from the interferon induced 1A promoter [14]. In pre-messenger RNAs, the editing sites can be found in protein-coding sequences [7], [13], [15], [16], [17] or in non-coding sequences such as UTRs and introns [7], [9], [10], [11], [12]. Since the cell translation machinery recognizes Inosine as Guanosine, editing in coding sequences can recode an amino acid and therefore affect the protein structure and function [7], [13], [15], [16], [17]. Most editing sites are found in non-coding sequences; about 90% of them are located within *Alu* repetitive elements [7], [9], [10], [11], [12]. The presence of Inosines in non-coding sequences may influence multiple cellular processes such as RNA interference, microRNA biogenesis and function, RNA stability, RNA localization, chromatin structure and alternative splicing [18], [19], [20], [21], [22], [23]. The editing level in the brain is particularly high [24]. Several findings suggest an important role for RNA editing in the central nervous system [7], [15], [17]. Abnormal editing patterns were shown in CNS disorders including epilepsy, amyotrophic lateral sclerosis (ALS), brain ischemia, depression and brain tumors [7], [25], [26], [27], [28]. Abnormal behavior was demonstrated in C. elegans and D.melanogaster when the ADAR enzymes were knocked-out [29], [30].

Previous studies showed that there is a significant increase in editing levels of protein-coding regions during brain development and *in-vitro* neural differentiation [31], [32], [33]. It was shown that in mice, ADAR1 is crucial for embryonic development and that ADAR2 is vital for normal brain function [34], [35], [36], [37].

We show here that A-to-I RNA editing of *Alu* elements is high in hESCs and decreases during differentiation. In addition, we show that ADAR1 has a global effect on gene expression in hESCs and suggest a role for ADAR1 mediated-editing of Alu elements in the regulation of hESC differentiation.

## Results

### Low editing levels in protein-coding regions during spontaneous and neuronal differentiation of hESCs

**Figure 1:**
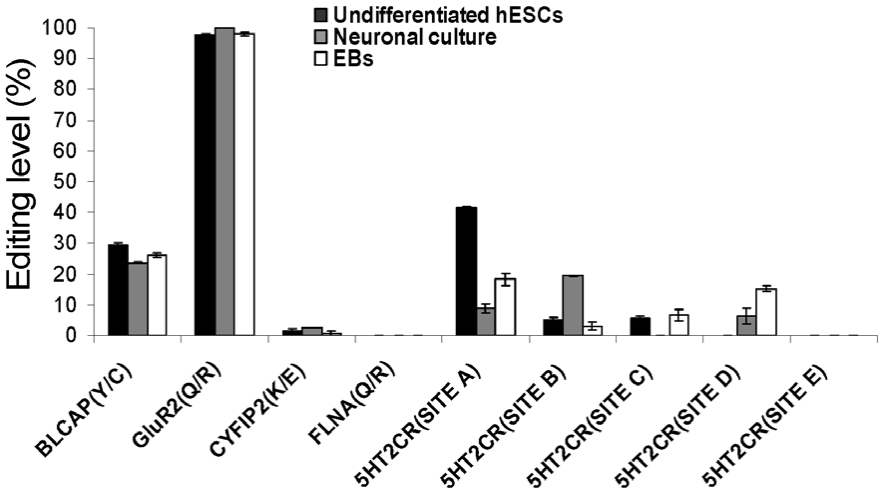
RNA editing within protein- coding regions during spontaneous and neuronal differentiation of hESCs. H9.2 hESCs were differentiated spontaneously or to highly enriched neuronal culture by EBs derivation or by using all-trans retinoic acid, respectively. The editing levels of BLCAP, CYFIP2, and FLNA mRNAs at sites: Y/C, K/E and Q/R, respectively, were measured by the SEQUENOME MassArray technology. The editing levels of 5HT2CR mRNA at sites: A, B, C, D and E and the editing level of GluR2 at site Q/R were measured by direct sequencing. Absence of bars means that editing is under the detection level.

Editing sites within protein-coding regions are relatively rare. Yet this type of RNA editing was shown to have a large effect on protein function and cell physiology [7], [15], [17]. In addition, editing within protein-coding regions was shown to be highly regulated during development and cell differentiation [31], [32], [33]. The editing levels in protein-coding regions of hESCs clonal line H9.2 were monitored during spontaneous differentiation or during neuronal differentiation (Figure 1 and Figure S1). RNA editing levels were measured by direct sequencing or SEQUENOM MassArray analysis. Editing was monitored in the coding region of five genes; GluR2 (Site Q/R), BLCAP (Site Y/C), 5HT2CR (Sites A-E), FLNA (Site Q/R) and CYFIP2 (Site K/E) (Figure 1 and references [15], [16], [17]). Significant editing levels were detected in GluR2, BLCAP and 5HT2CR (Sites A, C and D). GluR2 was almost 100% edited both before and after hESCs spontaneous or neuronal differentiation similar to the findings in adult brain. A modest decrease at the editing level of BLCAP was observed during neuronal differentiation (29.2%–23.6%). We observed a significant decrease in the editing level of 5HT2CR (Site A) during both neuronal differentiation and spontaneous differentiation (41.5%–8.8% and 41.5%–18.3%, respectively). In contrast, we observed a significant increase in the editing level of 5HT2CR (Site B) during the neuronal differentiation (5%–19.4%) and also of 5HT2CR (Site D), particularly, during the spontaneous differentiation (0%–15.2%). FLNA and CYFIP2 that are significantly edited in the adult [17], [38] were only marginally edited in undifferentiated, neuronal and EB cultures. Similarly, editing levels of 5HT2CR sites were much lower in hESCs and their derivatives compared to those observed in adult human brain [17].

Similar levels of editing were observed in the hESCs line I6 (data not shown). The low editing observed is in agreement with previous studies that showed increased editing levels within protein-coding regions during development [31], [32], [33]. In addition, the results show that there is no consistent pattern of editing regulation among protein-coding regions during hESCs neuronal and spontaneous differentiation.

### RNA editing levels within *Alu* sequences decrease during neuronal and spontaneous differentiations of hESCs

The presence of Inosines in non-coding sequences may influence multiple cellular processes related to RNA interference and microRNA biogenesis and function, RNA stability, RNA localization, chromatin structure and alternative splicing [18], [19], [20], [21], [22], [23]. Since, these processes are highly regulated during ESCs differentiation [39], [40], [41] it was of interest to monitor the levels of editing within non-coding RNA regions during hESCs differentiation (Figure S1).

The vast majority of A-to-I RNA editing events was found in non-coding *Alu* elements [7], [9], [10], [11], [12]. We monitored the editing levels of eight *Alu* sequences which have been shown to be edited in adult tissues. These *Alu* elements are embedded in 3′UTRs or introns of the following transcripts: C4orf29/hypothetical protein FLJ21106, F11R, FANCC, BRCA1, MDM4, RBBP9, CARD11 and THRAP1/MED13 ([9], [26] and data not shown). In order to monitor differences in the editing levels of differentiated and undifferentiated hESCs, we used the SEQUENOME MassArray technology or direct sequencing.

**Figure 2:**
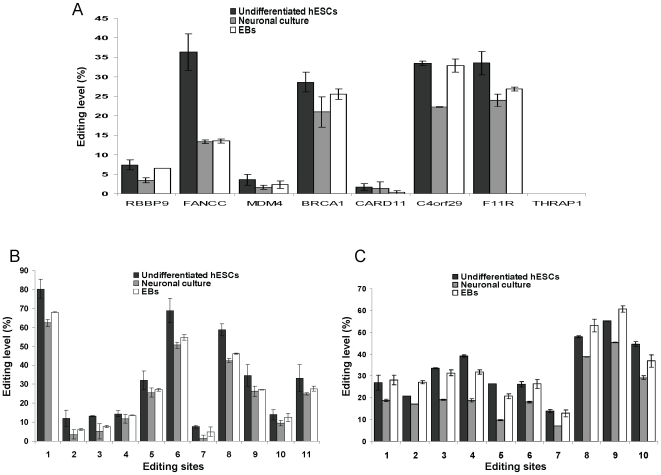
RNA editing levels within *Alu* sequences decrease during neuronal and spontaneous differentiation of hESCs. The editing levels within *Alu* sequences of eight genes during the differentiation of H9.2 hESCs were measured. (A) RNA editing levels of C4orf29, F11R and THRAP1 *Alu* sequences were measured by direct sequencing. C4orf29 editing levels represent the average of 10 sites and F11R editing levels represent the average of 11 sites. Editing levels within *Alu* sequences of: RBBP9, FANCC, MDM4, BRCA1 and CARD11 were measured by the SEQUENOME MassArray technology in specific discrete sites. No editing was detected in the *Alu* of THRAP1 (B, C) A detailed representation of editing levels of the *Alu* elements of C4orf29 and F11R in the undifferentiated hESCs, the neuronal culture and the EBs. Specific localization of each editing site is given in table S7.

All the *Alu* elements in the above transcripts were found to be edited except for THRAP1 (Figure 2A). The *Alu* sequences within FANCC, BRCA1, C4orf29 and F11R transcripts were edited at levels above 28% (Figure 2A). These findings indicate that *Alu* sequences are highly edited in both adult tissues as well as in hESCs. There was a significant decrease, from 20.7% to 12.4%, in the average editing level of *Alu* sequences as a result of neuronal differentiation (n = 7 edited *Alu* regions, p<0.016 according to Wilcoxon signed-rank test; Figure 2A). We observed also a significant decrease, from 20.7% to 15.4%, in the average editing level of *Alu* sequences in the spontaneous differentiation (n = 7 edited *Alu* regions, p<0.016 according to Wilcoxon signed-rank test; Figure 2A). Of note, the decrease in editing levels was consistent among different *Alu* sequences during neuronal differentiation (Figure 2A). In most *Alu* sequences the decrease in the editing level was less prominent during the spontaneous differentiation versus the neuronal differentiation (Figure 2A, B, C). In order to map and quantify all the clustered editing sites found in two representative genes, C4orf29 and F11R, they were monitored by direct sequencing. The analysis shows that during neuronal differentiation, the editing levels of the clustered sites were consistently decreased (Figure 2B, C). Taken together, these results suggest that decrease in *Alu* editing levels during neuronal differentiation is a global phenomenon. A similar trend was observed also during early spontaneous differentiation (After differentiation of 1–2 weeks) (Figure S2). A detailed analysis of the editing levels in C4orf29 and F11R during 3 weeks of spontaneous differentiation is shown in Figure S2.

### ADAR expression levels increase during neuronal and spontaneous differentiation of hESCs

**Figure 3:**
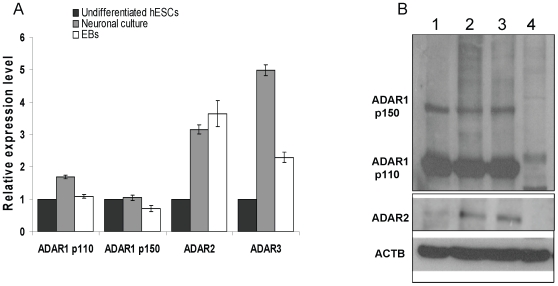
ADAR expression levels increase during neuronal and spontaneous differentiation of hESCs. (A) The relative expression levels of ADAR mRNAs during H9.2 hESCs differentiations. The analysis was performed by qRT-PCR. Normalization was performed by GAPDH. (B) Western blot analysis of ADAR proteins expression levels during H9.2 hESCs differentiations. lane1: Undifferentiated hESCs, lane2: Neuronal culture, lane3: EBs, lane4: MEFs. ACTB -beta Actin.

The alterations in editing levels we observed may be a result of differences in the expression of ADAR1-3 levels. In order to monitor changes in ADARs during differentiation, we compared the mRNA level of ADAR1-3 between H9.2 hESCs and their neuronal and EBs derivatives by qRT-PCR (Figure 3A and Figure S1). We detected three to five fold upregulation in mRNA expression levels of ADAR2 and ADAR3 as a result of differentiation (Figure 3A). A moderate upregulation in the mRNA levels of the ADAR1 p110 isoform was observed during neuronal differentiation but not during spontaneous differentiation. The mRNA level of the ADAR1 p150 isoform did not change significantly in both types of differentiation. In agreement with the fact that ADAR3 is brain specific in the adult and that ADAR1 p110 mRNA isoform is expressed at a relatively high level in the brain [7], [42], their expression levels in the neuronal culture were higher compared to EBs culture (2.17 and 1.55 folds, respectively; Figure 3A). In contrast, ADAR2 expression was similar in the neuronal and EBs cultures (Figure 3A).

Protein levels of the two ADAR1 isoforms were similar in hESCs, neuronal and EBs cultures (Figure 3B). In agreement with qRT-PCR data, ADAR2 protein expression levels in differentiated cultures were significantly higher compared to undifferentiated hESCs, without a significant difference between neuronal and EBs cultures (Figure 3B). The qRT-PCR data together with DNA microarray analyses indicate that the ADAR1 mRNA levels are significantly higher than those of ADAR2 and ADAR3 in undifferentiated hESCs (Figure S3).

The relative mRNA levels of ADAR1 and ADAR2 were also measured during spontaneous differentiation of the hESCs line I6 and compared with those of adult human cortex which is characterized by high editing levels (Figure S4). The regulation of ADAR mRNA expression level during differentiation was found to be largely similar in the two hESCs lines (H9.2 and I6) (Figure 3A and Figure S4). In addition, ADAR1 expression was only moderately lower in hESCs versus the cortex while ADAR2 expression was significantly lower (Figure S4). These results suggest that ADAR1 plays a significant role in hESCs at early stages of differentiation while ADAR2 and ADAR3 may be important at later stages.

### RNA editing levels of *Alu* sequences are highly dependent on ADAR1 in hESCs

**Figure 4:**
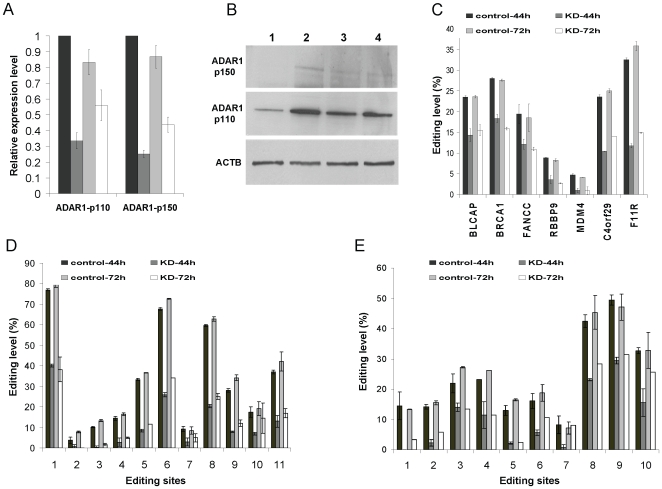
RNA editing levels of *Alu* sequences are highly dependant on ADAR1 in hESCs. H9.2 hESCs were grown under feeder-free conditions and transfected with ADAR1 siRNA or with non-target negative control siRNA. 44 h and 72 h after tranfection, total RNA and protein were derived for analysis. (A) The relative mRNAs expression levels of ADAR1 p110 and ADAR1 p150 from the siRNAs transfected hESCs. Relative expression levels were measured by qRT-PCR. Normalization was done using GAPDH. (B) Western blot analysis of ADAR1 p110 and ADAR1 p150 proteins after the siRNA transfections. Lane 1 and lane 2 refer to ADAR1-siRNA and non-target control siRNA transfected cultures after 44 h, respectively. Lane 3 and lane 4 refer to ADAR1-siRNA and control siRNA transfected cultures after 72 h, respectively. (C) The editing levels of BLCAP (Y/C) site and *Alu* sequences of six other genes in ADAR1 knockdown versus control hESCs. RNA editing levels of C4orf29 and F11R *Alu* sequences were measured by direct sequencing. C4orf29-*Alu* editing levels represent the average of 10 sites and F11R-*Alu* editing levels represent the average of 11 sites. Editing levels of the BRCA1, FANCC, RBBP9 and MDM4 tanscripts were measured by primer extension followed by mass spectrometry using the Sequenom MassArray in specific discrete sites. (D, E) A detailed representation of editing levels in the *Alu* sequences of C4orf29 and F11R in ADAR1 knockdown versus control hESCs which were measured by direct sequencing. Specific localization of each editing site is given in Table S7.

*Alu* sequences are members of the large SINEs (Short Interspersed Elements) family of repetitive elements which is found in all mammals. Recently, it was shown that ADAR1 is involved significantly in editing of SINE elements in mice [43]. In addition, we found that the ADAR1 mRNA level is significantly higher than that of ADAR2 and ADAR3 in hESCs (Figure S3). Therefore, we hypothesized that in hESCs ADAR1 has a substantial effect on RNA editing of *Alu* elements. To test this hypothesis, we specifically silenced ADAR1 expression by RNA interference. Undifferentiated cultures of hESCs were transfected with ADAR1 specific siRNA or with non-target siRNA as negative control and were subjected to qRT-PCR analysis. 44 h after transfection, the mRNA levels of the two ADAR1 isoforms, p110 and p150, decreased by 66% and 75% respectively, in cells treated by ADAR1-siRNA (Figure 4A). The silencing effect of p110 and p150 was decreased to 33% and 50%, respectively, 72 h after siRNA transfection (Figure 4A). The results were supported by western blotting. According to the western blot analysis, the ADAR1-p110 isoform was down-regulated 44 h after transfection. In contrast, no significant difference was observed after 72 h (Figure 4B). According to densitometry analysis normalized to beta actin, the ratios of p110 levels between knockdown and control were: 1∶5.53 after 44 h and 1.13∶1 after 72 h, respectively. ADAR1-p150 was also down-regulated 44 h after transfection (Figure 4B). The accurate quantification of ADAR1-p150 is not provided due to the very low expression level of this isoform.

In order to study the effect of decreased ADAR1 levels on the editing levels we used direct sequencing or the SEQUENOME MassArray technology for the quantification of editing. As expected, 44 h after transfection the editing level of BLCAP(Y/C) which is a specific target of ADAR1 [43] was 14.3% versus 23.5% in the control (Figure 4C). Both 44 h and 72 h after siRNAs transfection, ADAR1 knockdown cells had ∼two fold decrease in the average editing level of *Alu* elements (n = 6 *Alu* elements, p<0.04 according to Wilcoxon signed-rank test) (Figure 4C). It is important to note that the editing levels, 72 h after the ADAR1-siRNA transfection, remained low in spite of the fact that ADAR1 protein expression levels were increased and became the same as in the control. A possible explanation for this discrepancy can be that ADAR1 activity doesn't appear immediately after its translation because of unknown secondary processes which are required to mediate its editing function. Therefore, a large proportion of the RNAs remained unedited. Low editing levels after ADAR1-siRNA transfections were observed in two independent experiments, although ADAR1 expression level recovered after 72 hours and became equal to that of the control cells. To evaluate the knockdown effect on clustered editing sites within *Alu* sequences, the editing levels in F11R and C4orf29 were quantified by direct sequencing. This analysis showed that, both 44 h and 72 h after siRNA transfection, the ADAR1 silencing caused a global decrease in editing in the clustered editing sites within these *Alu* sequences (Figure 4D, E). Taken together, these data show that the editing levels of *Alu* sequences in hESCs are significantly dependent on ADAR1.

### Global effect of ADAR1 silencing on gene expression in hESCs

**Figure 5:**
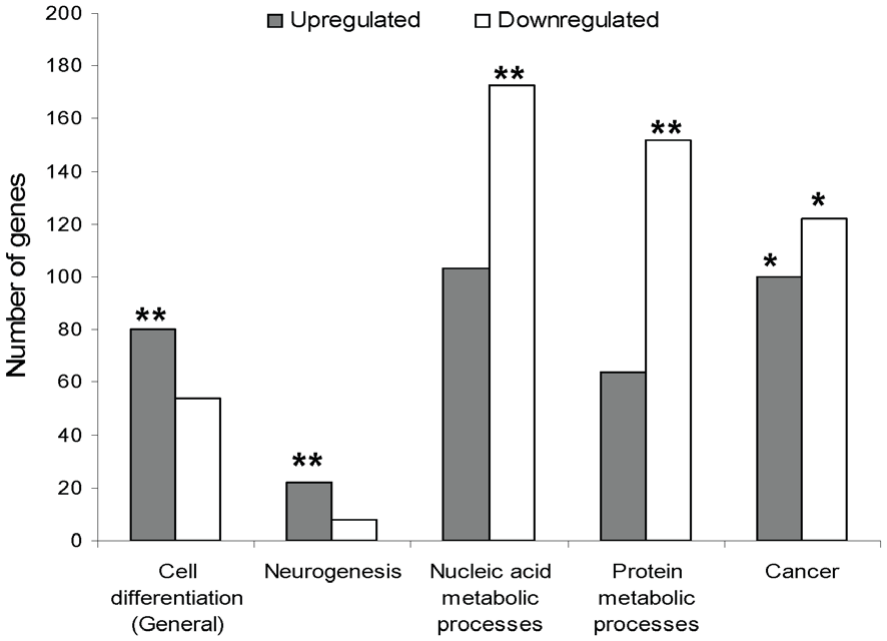
A global affect of ADAR1 silencing on gene expression in hESCs. 44 h after transfections, RNA were derived from hESCs treated with ADAR1-siRNA or with non-target siRNA negative control and analyzed by oligonucleotide Microarray. Annotated genes whose expression level was changed at least at 1.5 fold as a result of ADAR1 silencing were categorized into functional groups. Prominent functional groups of genes which were overrepresented in the upregulated and/or downregulated genes are shown. One star indicates p-value<0.05. Two stars indicate p-value<0.001.

RNA editing is a major post transcriptional modification that affects global gene expression and tends to occur at high levels in hESCs. We therefore speculated that it has a significant effect on the regulation of hESC differentiation. The finding that significant regulation of *Alu* editing occurs during hESC differentiation further supports that assumption. Therefore, we predicted that ADAR1 knockdown will have a significant effect on hESC global gene expression and phenotype. To study the effect of ADAR1 knockdown on gene expression, we performed microarray analysis using RNA extracted from the ADAR1 knockdown and the non-target control siRNA treated hESCs, 44 h after transfection. The expression levels of 867 annotated genes changed as a result of ADAR1 knockdown by at least 1.5 fold. Among them, 390 genes were upregulated and 477 genes were downregulated. The genes were clustered into functional groups by the DAVID Gene Functional Classification Tool (Table S1 and Table S2). Of particular interest were gene categories which are related to cell differentiation processes in general and to neurogenesis in particular. We found that both groups are significantly overrepresented among the upregulated, but not among the downregulated genes (p<0.0001 and p>0.8, respectively; Figure 5, Table 1, Table S1 and Table S2). Interestingly, that trend is also evident for most other functional groups of genes which are related to cell differentiation/development processes (Tables S1 and S2). Among the downregulated genes, those related to nucleic acid metabolism and protein processing were significantly overrepresented (Figure 5, Table 1, Table S1 and Table S2). In addition, according to Ingenuity IPA software, among the functional groups the cancer category has the largest number of genes whose expression level was affected by ADAR1 knockdown. Both upregulated and downregulated cancer related genes were significantly overrepresented (p<0.05; Figure 5, Table 1 and Table S3).

**Table 1:** Global effect of ADAR1 silencing on gene expression in hESCs.

	Upregulated genes		Downregulated genes	
Type of functional group	Number of genes	P-value of overrepresentation	Number of genes	P-value of overrepresentation
Cell differentiation (general)	80	0.0000077	54	0.84
Neurogenesis	22	0.000094	8	0.88
Nucleic acid metabolic processes	103	0.76	173	0.000026
Protein metabolic processes	64	1.0	152	0.00067
Cancer	100	<0.05	122	<0.05

44 h after transfections, RNA were derived from hESCs treated with ADAR1-siRNA or with non-target siRNA negative control and analyzed by oligonucleotide Microarray. Annotated genes whose expression level was changed at least at 1.5 fold as a result of ADAR1 silencing were categorized into functional groups. Prominent functional groups of genes which were overrepresented in the upregulated and/or downregulated genes are shown.

Differentiation of ESCs into cells of the three germ layers is characterized by a decrease at the mRNA level of genes such as two of the pluripotency master regulators Oct4 and Nanog and in significant changes of their specific morphology [2], [44], [45], [46]. We didn't observe differences at the mRNA level of Oct4 and Nanog. (Figure S5A). In addition, cells after siRNA transfection with either ADAR1 specific or non-target control siRNA (44 h and 72 h) didn't exhibit significant morphological changes (Figure S5B).

Taken together, we conclude that ADAR1 is not essential for maintenance of hESCs undifferentiated state, at least not in the short term.

Although ADAR1 silencing didn't affect hESCs status under our experimental conditions (cells were grown under culture conditions which are designed to inhibit cell differentiation), the expression level of genes related to differentiation/developmental processes increased. Therefore, we suggest that differentiation of ADAR1 knockdown cells may be accelerated under conditions which support differentiation.

## Discussion

Previous findings on RNA editing in the context of mammalian development are based on animal models or on the analysis of human tissues at different stages of development. These studies showed that RNA editing levels in protein-coding regions increase significantly during brain development [31], [32] and that the ADAR enzymes are important for normal development [34], [35], [36], [37]. However, the relevance of animal models to the role of RNA editing in human development is limited since it was shown by our previous work that RNA editing level in humans is much higher than that of laboratory animals [38]. In addition, the reported studies of editing in human tissues analyzed only samples taken after mid-gestation when most of the differentiation processes had already been completed. Furthermore, previous studies regarding RNA editing in development analyzed only editing sites within protein-coding regions and did not address the vast majority of editing events which occur within non-coding regions. Therefore, the relevance of these studies to cell differentiation research is limited. In the current study, we characterized RNA editing in both coding and non-coding RNA sequences in hESCs and their differentiated progenies, which provide a good model for research of early cell differentiation processes. As there is a significant increase in editing within protein-coding sequences during development, it was expected that RNA editing levels are low in human blastocysts and therefore also in its hESC derivatives. In addition, it was predicted that differentiation of hESCs, particular to the neural lineage, will be accompanied by increased RNA editing. Interestingly enough, these predictions were contradicted by our results: We found significant editing levels of *Alu* sequences in undifferentiated hESCs and observed that differentiation, particularly to the neural lineage, was accompanied by decreased editing levels. In addition, no consistent pattern of editing regulation among protein-coding regions was observed. Therefore, it can be suggested that the widespread RNA editing in non-coding regions has a significant role in hESC early differentiation. The high levels of editing of non-coding sequences in hESCs and the decrease in global editing during differentiation suggest that the presence of Inosines in hESC transcripts may have significant effects on cellular regulatory mechanisms such as microRNA biogenesis and function, regulation of mRNA localization and stability, chromatin structure and alternative splicing which are known to be highly regulated during hESC differentiation [39], [40], [41].

mRNAs containing hyperedited inverted repetitive elements in their 3' untranslated regions (3'UTRs) are retained in nuclear paraspeckle-associated complexes containing the proteins p54^nrb^, PSF, and PSP1alpha [21], [47]. Chen et al reported recently that 3′UTR edited sequences in hESCs escape nuclear retention [47]. They showed that paraspeckles are absent from hESCs and only appear upon differentiation. In addition they showed that paraspeckle assembly and function depend on expression of a long nuclear-retained non-coding RNA, NEAT1, which is not detectable in hESCs. These findings, together with our data suggest that non-coding RNA editing has different roles in hESCs versus their differentiated derivatives.

Using RNAi silencing we have shown that *Alu*-sequence editing in hESCs is highly dependant on ADAR1 expression level. Yet, the control of global editing within repetitive element sequences during hESC differentiation is more complex. We show here that ADAR1 expression level is not significantly changed and that ADAR2 and ADAR3 expression increased significantly as result of hESC differentiation while the editing levels of *Alu* sequences decreased. The discrepancy may be explained in part by the possible role of ADAR3, whose expression level increased significantly mainly during neural differentiation, as a possible inhibitor of ADAR1 activity [48]. Other unknown factors which are involved in regulation of editing processes may also change during hESCs differentiation in a manner which affects global editing patterns.

We demonstrate here that silencing of ADAR1 leads to decreased global editing levels of *Alu* sequences in hESCs and results in significant induction of the expression of genes related to development and differentiation. However, we showed that expression levels of pluripotency master regulator genes, specifically Nanog and Oct4, were not changed under ADAR1 knockdown conditions and that the hESC morphology was compatible with the undifferentiated state. Our conclusion is therefore that ADAR1 is not essential for maintenance of the undifferentiated state of hESCs, at least in the short term.

Recently, it was demonstrated that ADAR1 is essential for the maintenance of hematopoiesis and suppression of interferon signaling [49]. The authors note that ADAR1 is dispensable for mouse ESC self-renewal and EB formation, in accordance with our findings in the human model. In addition, some studies suggest that high expression levels of differentiation genes are not sufficient to interfere with the ESC undifferentiated state, possibly because ESC undifferentiated status depends mainly on the their master regulator genes. For example, these studies showed that when expression levels of many genes, related to diverse differentiation processes, were increased by interfering with the Polycomb group proteins, the undifferentiated state of the ESCs continued to be maintained and the expression levels of the master regulators remained high [50], [51], [52]. Similarly, editing may not be essential for maintenance of hESC undifferentiated state. However, since ADAR1 silencing results in an increase in the expression levels of genes which are related to differentiation/developmental processes, we speculate that it may have a role in the regulation of early cell differentiation processes.

Bioinformatics analysis suggests that neighboring, reversely oriented *Alu* elements within 3′UTRs, are often cleaved at both ends of the region harboring the inverted repeats followed by rejoining of the two parts of the transcript. These shortening events are more common in tissues characterized by high editing levels [53]. Therefore, editing may induce shortening of 3′UTRs. Recently, the 3′UTRs of induced pluripotent stem (iPS) cells, which have many of the characteristics of ESCs, were shown to be shorter than those of their somatic cell of origin. The mechanism suggested for the shortening is alternative polyadenylation [54], similar to the mechanism implicated in 3′UTR shortening in activated T cells and cancer cells [55], [56]. Similarly Prasanth et al implicated alternative polyadenylation in the shortening of the mouse Slc7a2 3′ UTR, resulting in removal of the hyperedited sequences involved in anchoring to the nuclear parascpeckles [57]. We would like to suggest an additional mechanism whereby the high level of editing in undifferentiated embryonic stem cells may be related to the cleavage and shortening of 3′UTR sequences in such cells.

We can not rule out the possibility that other editing independent functions of ADAR1 [58], [59], [60] mediated the increased expression level of cell differentiation/development related genes. However, our finding that *Alu* editing levels decrease significantly during hESC differentiation, without a decrease in ADAR1 expression levels, suggests that those alterations are indeed editing dependent.

We and others showed that A-to-I RNA editing levels in various solid tumors are significantly lower in comparison with the corresponding normal tissues and that overexpression of ADARs in brain tumor cell lines resulted in decreased proliferation and migration rates [26], [27]. These findings suggest that decreased editing levels may contribute to tumorigenesis and cancer pathogenesis. Cancer stem cells share a lot of features with normal stem cells [61], [62].Our findings here describing the significant alteration of expression of cancer related genes by ADAR1 knockdown in hESCs further support the role of dysregulated editing in cancer (Table S3).

In conclusion, our findings show that ADAR1 has a global influence on the expression level of many genes that are related to development processes, cancer, nucleic acid and protein processing etc. This effect is probably mediated by the high editing levels of *Alu* elements that appear in undifferentiated hESCs. Our data strongly suggest that upon differentiation, *Alu* editing levels decrease in a manner that may play a role in the regulation of hESC early differentiation decisions.

## Materials and Methods

### Cell culture

hESCs (H9.2 and I6 [63], [64]) were cultured on a mitomycyin C-treated mouse embryonic fibroblasts (MEFs) feeder layer obtained in medium consisting of 80% KnockOut DMEM, 20% KnockOut serum replacement, 1 mM L-Glutamine, 0.1 mM β-mercaptoethanol, 1% non essential amino acids stock solution (×100), 4 ng/ml basic fibroblast growth factor (All from Gibco) and Penicillin (50 units/ml)/Streptomycin (50 µg/ml) (Biological Industries, Israel). To obtain feeder-free culture, hESCs were grown on growth factor reduced Matrigel (BD Biosciences) coated plates with MEFs conditioned medium [65]. *In-vitro* differentiation into Embryoid Bodies (EBs) was performed by transferring hESCs colonies to low attached Petri dishes (Miniplats, Ein-Shemer, Israel) and growing them in medium consisting of 80% KnockOut DMEM, 20% defined Fetal Bovine Serum (HyClone), 1 mM of L-Glutamine, 1% of non essential amino acids stock solution (×100) and Penicillin (50 units/ml)/Streptomycin (50 µg/ml). To differentiate hESCs into neuronal cells, EBs were treated according to Shuldiner et al [66] with few changes. In brief, after 4 days in suspension, 5 µM all-trans retinoic acid (Sigma-Aldrich) were added to the EBs medium. The medium was replaced every 2 days. After 21 days in suspension, cells were seeded on rat tail collagen (Roche) in the same medium for an additional 2–3 days.

### RNA purification and reverse transcription

Total RNA was isolated using Trizol reagent (Invitrogen) according to the manufacturer's instructions. Random-primed cDNA synthesis was done on 1 µg -2 µg of total RNA using M-MLV reverse transcriptase (Promega) according to manufacturer's instructions.

### Quantitative Real-Time PCR (qRT-PCR)

qRT-PCRs were performed on a 7900HT ABI platform using 2X SYBR Green master mix (ABI). The relative mRNAs expression levels analyses were done by the 2^−ΔΔCT^ method with GAPDH as the internal control for normalization. Data analysis was performed using the SDS 2.1 Software (ABI). Primers are listed in table S4.

### Western blot

For protein derivation, cells pellets were lysed on ice by RIPA lysis buffer consisting of 150 mM NaCl, 1% NP40, 0.5% sodium deoxycholate, 0.1% SDS and 50 mM Tris-HCl PH 8 supplemented with protease inhibitor (Roche). Alternatively, proteins were isolated from Trizol homogenates according to the manufacturer's instructions. Proteins were denaturated in sample buffer, resolved on SDS-PAGE gel and transferred to nitrocellulose membranes according to standard protocols. Following blocking with 5% dry milk in Tris-buffered saline containing 0.05% Tween 20, the membranes were stained with the following antibodies: Polyclonal anti ADAR1 (Generous gift from Brenda Bass), polyclonal anti ADAR2 (Generous gift from Marie Öhman) or polyclonal anti Beta-Actin (Santa Cruz). Specific reactive bands were detected using goat anti-rabbit (ADAR1 and ADAR2) or donkey anti goat (Beta-Actin) conjugated to horseradish peroxidase (Jackson Laboratory). Immunoreactive bands were visualized by the SuperSignal West Pico Substrate Chemiluminescent kit (Pierce).

### Immunofluorescence staining

Cells were fixed with 4% paraformaldehyde for 25 minutes.

Oct4 staining: Cells were permeabilized and blocked in room temperature in 2 stages: Cells were incubated for 10 minutes with PBS containing 1% Triton X-100 (Sigma); Cells were then incubated for a further 15 minutes with PBS supplemented with 1% Triton X-100 and 2% normal goat serum (Invitrogen).

Cells were incubated for 2 hours with mouse monoclonal Oct4 antibody (Chemicon) at a dilution of 1∶20 in PBS supplemented with 1% Triton X-100 and 2% normal goat serum.

Tubulin, beta 3 staining: Cells were permeabilized and blocked for 30 minutes in a solution of PBS supplemented with 3% bovine serum albumin (Sigma-Aldrich), 0.3% Triton X-100, and 5% normal goat serum. The cells were incubated for 2 hours with mouse monoclonal anti tubulin, beta 3 antibody (Sigma-Aldrich) at a dilution of 1∶1000 in the same solution.

Both stains were visualized by goat anti mouse Alexa Fluor 488 (Invitrogen, Molecular Probes) at a concentration of 1∶1000. Nuclear staining was performed by DAPI (Sigma).

### Editing analysis by direct sequencing

PCR amplifications of known edited regions were done using PCR Master Mix (Thermo Scientific). The resulting PCR-fragments were purified using HiYield gel purification kit (RBC), and sequenced using ABI Prism 3100 genetic analyzer (ABI). The levels of editing assessed by sequencing were quantified by the DS Gene program (Accelrys). Primers are listed in table S5.

### Editing quantification by the SEQUENOME MassArray technology

RNA editing detection was carried out using MALDI-TOF mass spectrometer (Sequenom, San Diego, CA) as described for mutation detection [67]. In brief, for each editing site two specific primers flanking the editing site and one extension primer complementary to a sequence adjacent to the editing site were designed using MassARRAY assay design software (Sequenom). Following amplification of the region of interest, a primer extension reaction was carried out. This reaction included sequence specific hybridization and sequence dependent termination that generated different products for the non-edited and edited cDNA fragments, each with its unique mass values. RNA editing levels at specific sites were determined by spotting the extension products onto silicon chips preloaded with proprietary matrix (SpectroChip; Sequenom) that were subsequently read by the MALDI-TOF mass spectrometer. Primers are listed in table S6.

### RNA interference

hESCs were plated at 2×10^5^ cells per well in a six-well plate and were grown under feeder-free conditions. 24 hours later cells transected with ADAR1-siRNA (ID: 119581, Ambion) or with non-target siRNA control (AM4611- Negative control - #1, Ambion) using Lipofectamine 2000 (Invitrogen). Lipofectamine - siRNA complexes were produced in Opti-MEM I medium (Invitrogen) according to the manufacturer protocol. Transfections were done with a medium consisting of 2 ml MEFs conditioned media and 1 ml Opti-MEM I containing 10 µl Lipofectamine and 200 pmol siRNA per well.

### Oligonucleotide Microarrays

All experiments were performed using Affymetrix HU GENE1.0st oligonucleotide. Total RNA from each sample was used to prepare biotinylated target DNA, according to manufacturer recommendations. Briefly, 100–600 ng of Total RNA was used to generate first-strand cDNA by using a T7-random hexamers primer. After second-strand synthesis, in vitro transcription was performed. The resulting cRNA was then used for a second cycle of first-strand cDNA by using a T7-random hexamers primer with UTP resulting in SS DNA used for fragmentation and terminal labeling. The target cDNA generated from each sample was processed as per manufacturer's recommendation using an Affymetrix GeneChip Instrument System. Briefly, spike controls were added to 5.5 µg fragmented cDNA before overnight hybridisation. Arrays were then washed and stained with streptavidin-phycoerythrin, before being scanned on an Affymetrix GeneChip scanner. The quality and amount of starting RNA was confirmed using Bioanalyser (Agilent). After scanning, array images were assessed by eye to confirm scanner alignment and the absence of significant bubbles or scratches on the chip surface.

Genes were analyzed using unsupervised hierarchical cluster analysis (Spotfire DecisionSite for Functional Genomics; Somerville, MA) and were filtered according to fold change calculation. DAVID and Ingenuity IPA software (http://david.abcc.ncifcrf.gov/, http://www.ingenuity.com/) were used for gene functional classification and overrepresentation calculations. All data is MIAME compliant and the raw data has been deposited in the Gene Expression Omnibus (GEO) (http://www.ncbi.nlm.nih.gov/geo) as GSE 19719 (temporary number).

## Supporting Information

Figure S1 Differentiation of hESCs. H9.2 hESCs were transferred to suspension growth conditions and grown in EB medium in order to induce differentiation (Day 0). On day 5, all-trans retinoic acid was added to the medium in order to support mainly neural differentiation. The cells in suspension were grown with retinoic acid during 17 days. As control for the retinoic acid supportive influence on neuronal enrichment and for the induction of spontaneous differentiation, a second group of cells were grown without retinoic acid (EBs). In order to estimate the neuron frequency, the aggregates were dissociated by trypsinization and seeded on rat tail collagen where they were grown for an additional 2–3 days. (A) A typical hESCs colony growing in co-culture with inactivated MEFs. Scale bar: 10 µm. (B, C) Immunofluorescence staining of the hESCs specific protein, Oct4 (green), before the differentiation treatment. DAPI staining (Blue). Scale bar: 100 µm. (D,E). On day 22, the aggregates of the retinoic acid treated group (D) were significantly less cystic than the EBs (E). Scale bar: 1000 µm. (F,G) 3 days after seeding the cells on collagen, most of the retinoic acid treated cells had a typical neuronal morphology and were organized in a net like structures (F). In contrast, in the culture of the seeded EBs, only a minority of the cells had a neuronal morphology (G). Scale bar: 100 µm. (H,I) Immunostaining images demonstrating the expression of beta, 3 tubulin (TUBB3), a neuronal specific marker, in a large fraction of the retinoic acid treated cells (H). In contrast, a much smaller cells fraction expressed the TUBB3 protein in the EBs culture (I). Scale bar: 240 µm. Green refers to TUBB3 and blue refers to DAPI staining. (J,K) mRNA relative expression levels of undifferentiated hESCs markers and neuronal markers were measured in undifferentiated hESCs, retinoic acid treated cells and seeded EBs by qRT-PCR. This data shows a significant decrease at the expression level of the pluripotency markers, Oct4 and Nanog, after the differentiation treatments (J). In addition there is a significant increase at the expression level of the neuronal markers, NF68, TUBB3, MAP2, ELAVL3 and NFH after retinoic acid treatment (K). Measurments were normalized to GAPDH.
(0.77 MB TIF)

Figure S2 Editing levels of *Alu* sequences during EBs differentiation. H9.2 hESCs were differentiated spontaneously by EBs derivation. Total RNA were derived from the undifferentiated hESCs and the EBs after 1 week, 2 weeks and 3 weeks of growth in suspension. Editing levels of F11R-*Alu* (A) and C4orf29-*Alu* (B) sites are presented. Specific sites localization is presented in table S7.
(0.69 MB TIF)

Figure S3 The expression level of ADAR1 RNA in hESCs. The expression level of ADAR1 RNA in hESCs is significantly higher than those of ADAR2 and ADAR3. (A) Microarray signal intensity of ADAR1, ADAR2 and ADAR3 RNAs in undifferentiated H9.2 hESCs. (B) Real time PCR amplification plots of ADARs mRNA in H9.2 undifferentiated hESCs.
(2.18 MB TIF)

Figure S4 mRNA expression level of ADAR1 and ADAR2 during spontaneous differentiation of I6 hESCs. I6 hESCs were differentiated to EBs. The relative expression levels of ADAR1 (Common region of both p110 and p150 isoforms) and ADAR2 mRNAs were measured by qRT-PCR at different times during the differentiation, and in adult human cortex.
(0.60 MB TIF)

Figure S5 ADAR1 knockdown in hESCs resulted in similar expression levels of pluripotency markers and cell morphology. (A) qRT-PCR analysis of Oct4 and Nanog. Relative mRNA levels revealed similar levels in transient ADAR1 knockdown and control hESCs. (B) Light microscopy images of transient ADAR1 knockdown and control hESCs, 72 h after siRNAs transfections, revealed similar cell and colony morphology.
(4.22 MB TIF)

Table S1 Downregulated genes after ADAR1 knockdown. 44 h after transfections, RNA were derived from H9.2 hESCs that were treated with ADAR1-siRNA or with non-target siRNA negative control and analyzed by DNA Microarray. Annotated genes whose expression level was changed at least 1.5 fold as a result of ADAR1 silencing were categorized into functional groups by DAVID Gene functional Classification Tool.
(0.05 MB XLS)

Table S2 Upregulated genes after ADAR1 knockdown. 44 h after transfections, RNA were derived from H9.2 hESCs that were treated with ADAR1-siRNA or with non-target siRNA negative control and analyzed by DNA Microarray. Annotated genes whose expression level was changed at least 1.5 fold as a result of ADAR1 silencing were categorized into functional groups by DAVID Gene functional Classification Tool.
(0.07 MB XLS)

Table S3 Cancer related genes that were regulated after ADAR1 knockdown. This table represents cancer related genes whose expression level was changed at least 1.5 fold as a result of ADAR1 silencing. Analysis was performed using Ingenuity IPA software.
(0.02 MB XLS)

Table S4 List of primers used for qRT-PCR.
(0.03 MB DOC)

Table S5 List of primers used for direct sequencing. Forward and reverse primers were used for the amplification of the edited region. The reverse primers were used as the sequencing primers.
(0.03 MB DOC)

Table S6 List of primers used for SEQUENOME MassArray analysis.
(0.04 MB DOC)

Table S7 Localization of editing sites within *Alu* sequences (Mar. 2006 assembly (UCSC)).
(0.02 MB XLS)
