# Supplementary figures and images for: Alu Sequences in Undifferentiated Human Embryonic Stem Cells Display High Levels of A-to-I RNA Editing
Source: PLoS One. 2010 Jun 21;5(6):e11173. doi: 10.1371/journal.pone.0011173 (PMC2888580; doi:10.1371/journal.pone.0011173)

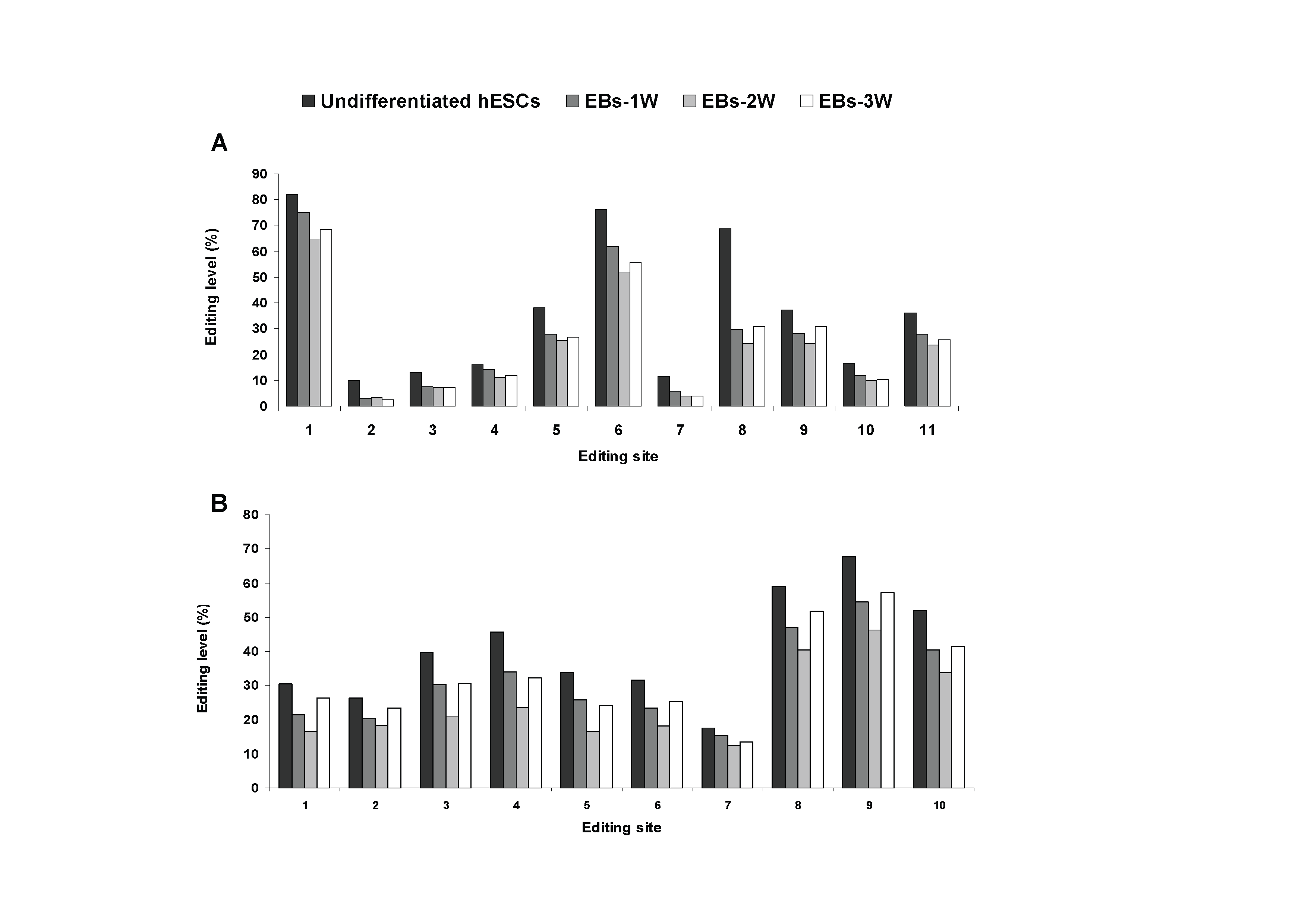

Supplement: Figure S2 — Editing levels of Alu sequences during EBs differentiation. H9.2 hESCs were differentiated spontaneously by EBs derivation. Total RNA were derived from the undifferentiated hESCs and the EBs after 1 week, 2 weeks and 3 weeks of growth in suspension. Editing levels of F11R-Alu (A) and C4orf29-Alu (B) sites are presented. Specific sites localization is presented in table S7. (0.69 MB TIF) [file pone.0011173.s002.tif]

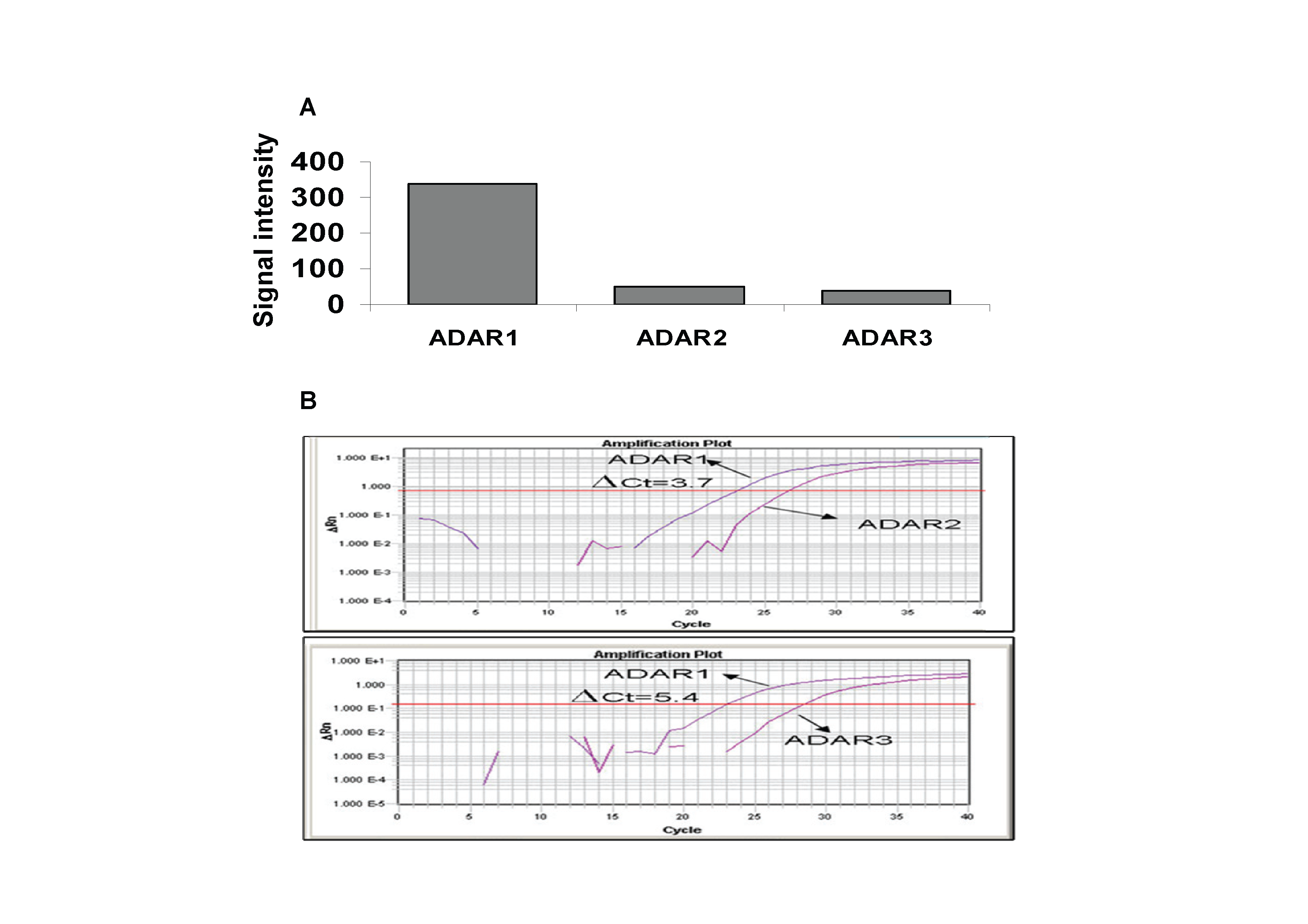

Supplement: Figure S3 — The expression level of ADAR1 RNA in hESCs. The expression level of ADAR1 RNA in hESCs is significantly higher than those of ADAR2 and ADAR3. (A) Microarray signal intensity of ADAR1, ADAR2 and ADAR3 RNAs in undifferentiated H9.2 hESCs. (B) Real time PCR amplification plots of ADARs mRNA in H9.2 undifferentiated hESCs. (2.18 MB TIF) [file pone.0011173.s003.tif]

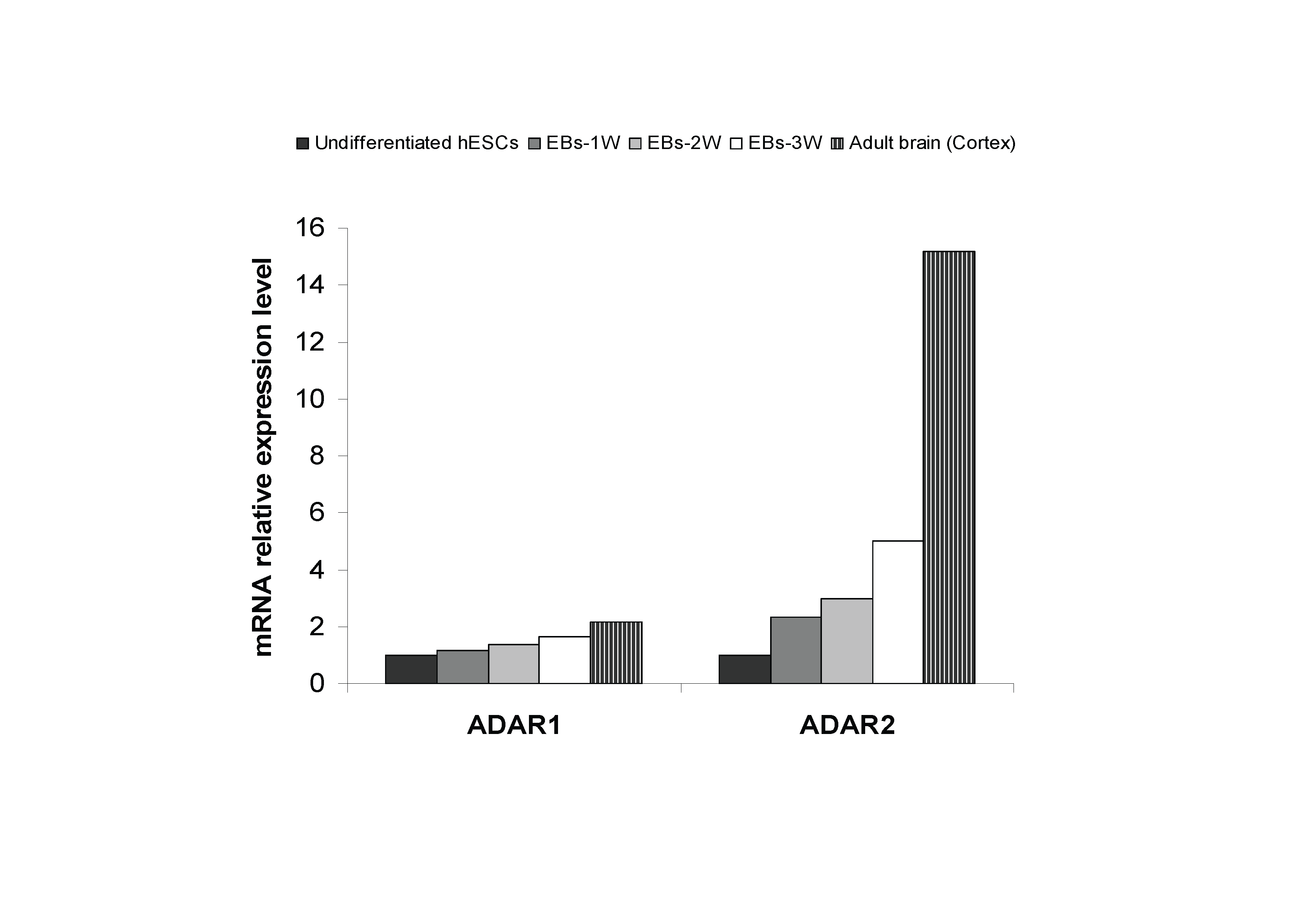

Supplement: Figure S4 — mRNA expression level of ADAR1 and ADAR2 during spontaneous differentiation of I6 hESCs. I6 hESCs were differentiated to EBs. The relative expression levels of ADAR1 (Common region of both p110 and p150 isoforms) and ADAR2 mRNAs were measured by qRT-PCR at different times during the differentiation, and in adult human cortex. (0.60 MB TIF) [file pone.0011173.s004.tif]

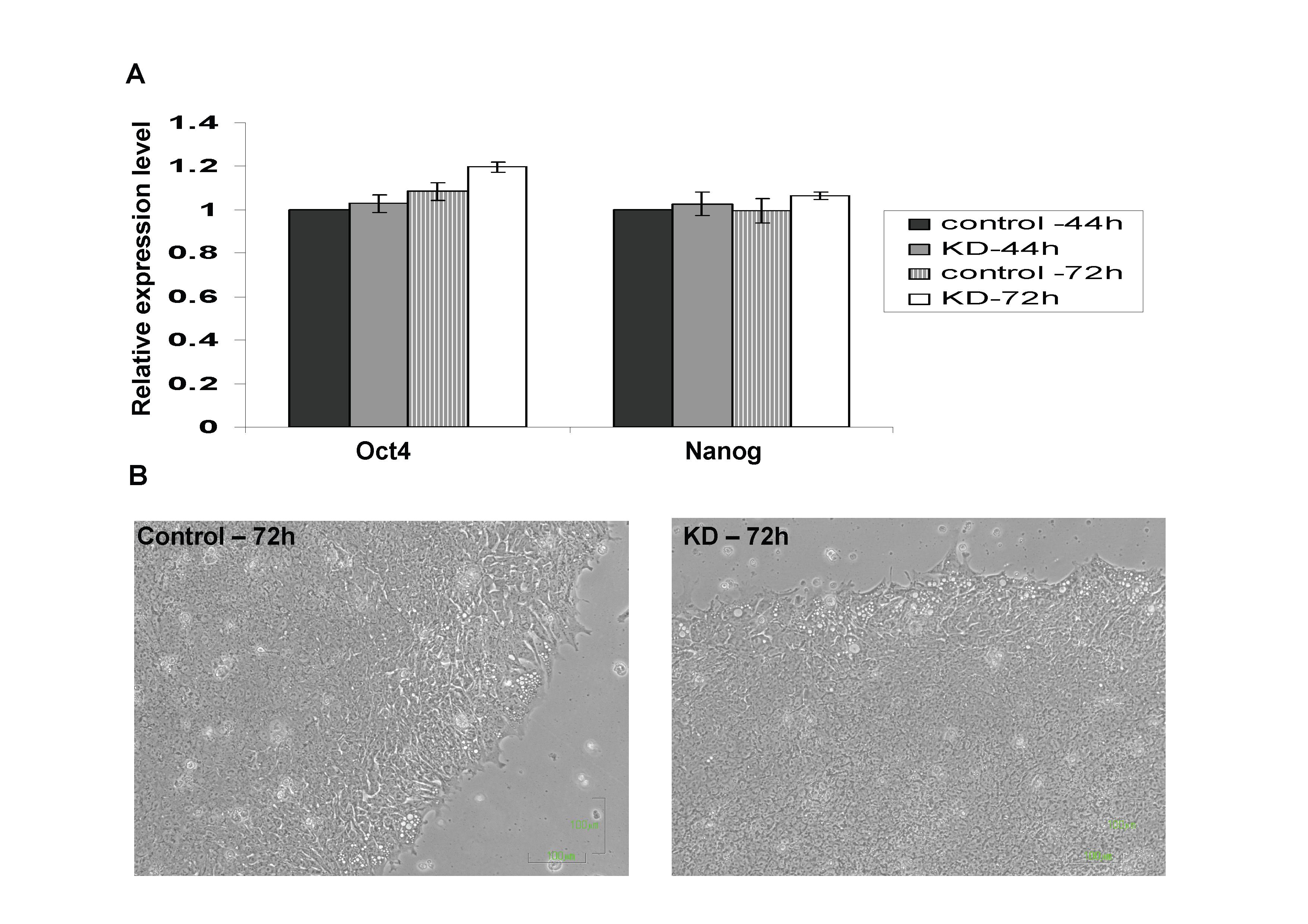

Supplement: Figure S5 — ADAR1 knockdown in hESCs resulted in similar expression levels of pluripotency markers and cell morphology. (A) qRT-PCR analysis of Oct4 and Nanog. Relative mRNA levels revealed similar levels in transient ADAR1 knockdown and control hESCs. (B) Light microscopy images of transient ADAR1 knockdown and control hESCs, 72 h after siRNAs transfections, revealed similar cell and colony morphology. (4.22 MB TIF) [file pone.0011173.s005.tif]
