# Supplementary material for: Alu Sequences in Undifferentiated Human Embryonic Stem Cells Display High Levels of A-to-I RNA Editing
Source: PLoS One. 2010 Jun 21;5(6):e11173. doi: 10.1371/journal.pone.0011173 (PMC2888580; doi:10.1371/journal.pone.0011173)
Supplement: Table S4 — List of primers used for qRT-PCR. (0.03 MB DOC) [file pone.0011173.s009.doc]

**Table S4. Primers used for qRT-PCR analyses**

| **Gene name** | **Forward Primer** | **Reverse Primer** |
| --- | --- | --- |
| ADAR1 – P110 (1B) | 5'GGCAGCCTCCGGGTG3' | 5'CTGTCTGTGCTCATAGCCTTGA 3' |
| ADAR1 – P150 | 5'CGGGCAATGCCTCGC3' | 5'AATGGATGGGTGTAGTATCCGC3' |
| ADAR2 | 5'CCGCAGGTTTTAGCTGACG3' | 5'CGGTCAGGTCACCAAACTTACC3' |
| ADAR3 | 5'CCCGCCATAAAGCGCTG  3' | 5'CCGAGCATCCAGGCCTT3' |
| Oct4 | 5'CCACACTGCAGCAGATCAGC3' | 5'GAACCACACTCGGACCACATC3' |
| Nanog | 5'TGAACCTCAGCTACAAACAG GTG3' | 5'GGTGCTGAGGCCTTCTGC3' |
| NF68 | 5'CGCTATGCAGGACACGATCA3' | 5'GGTATCGTGCCATTTCACTCTTT3' |
| NFH | 5'TAGCCGCTTACAGAAAACTCCTG3' | 5'AAGCGAGAAAGGAATTGGGC3' |
| MAP2 | 5'CAAAGAGAATGGGATCAACGG3' | 5'TCCTTGCAGACACCTCCTCTG3' |
| TUBB3 | 5'GGCGACTCGGACTTGCAG3' | 5'AGGCACGTACTTGTGAGAAGAGG3' |
| ELAVL3 ( HU-ANTIGEN C) | 5'TCTTCTCCCAGTACGGCCG3' | 5'ACCCCGAGAGACACCTGTGAC3' |
| GAPDH | 5'CCACATCGCTCAGACACCAT3' | 5'GGCAACAATATCCACTTTACCAG3' |
