# Supplementary material for: Alu Sequences in Undifferentiated Human Embryonic Stem Cells Display High Levels of A-to-I RNA Editing
Source: PLoS One. 2010 Jun 21;5(6):e11173. doi: 10.1371/journal.pone.0011173 (PMC2888580; doi:10.1371/journal.pone.0011173)
Supplement: Table S5 — List of primers used for direct sequencing. Forward and reverse primers were used for the amplification of the edited region. The reverse primers were used as the sequencing primers. (0.03 MB DOC) [file pone.0011173.s010.doc]

**Table S5. Primers used for direct sequencing analyses**

| **Gene** | **Forward primer** | **Reverse primer** |
| --- | --- | --- |
| F11R | 5'CCAAAAGGATTTA  AAACCGCTGC3' | 5'GAGCTGGAGTTTTGCT  CTTGTTGC3' |
| C4orf29 | 5'CTGTCTAGAACAGT  GCCTGATAAATAATAAGC  3' | 5'GGAATGCCATGGTGT  GATCTTG3' |
| THRAP1 | 5'GATCTTTTAAGGCTTG  TTTCTAAGAC3' | 5'GTCAGCAGGTCTCTT  GAAAG3' |
| 5-HT2CR | 5'CTGTCTCTCCTGGCAA  TCCTT3' | 5'GTCCCTCAGTCCAATC  ACAGG3' |
| GluR2 | 5'ttgcctacattggggtcagt3' | 5'actttcgatgggagacacca3' |
