# Supplementary material for: Alu Sequences in Undifferentiated Human Embryonic Stem Cells Display High Levels of A-to-I RNA Editing
Source: PLoS One. 2010 Jun 21;5(6):e11173. doi: 10.1371/journal.pone.0011173 (PMC2888580; doi:10.1371/journal.pone.0011173)
Supplement: Table S6 — List of primers used for SEQUENOME MassArray analysis. (0.04 MB DOC) [file pone.0011173.s011.doc]

**Table S6. Primers used for sequenome MassArray analyses**

| **Gene** | **Forward primer for PCR amplification of the edited region** | **Reverse primer for PCR amplification of the edited region** | **Extension primer** |
| --- | --- | --- | --- |
| BLCAP | 5' ACGTTGGATG-ATTAGGTCGGTTCCTGCAGCACGTTGGATGATTAGGTCG | 5'ACGTTGGATG-AGGAGGACGGGCA  GCAACGTTG GATGAGGAGGAC | 5'GCAGCCACTGGAGGCAA |
| CYFIP2 | 5'ACGTTGGATG-TTTCGGCGACATGCAGATAG | 5'ACGTTGGATG-ACGTCCACTTGGACTTGTTC | 5'CTCTTCATAGTGAGCACTGGTCT |
| FLNA | 5'ACGTTGGATG-TTCGTGGTGCCTGTGGCTT | 5'ACGTTGGATG-TGGGCGGTTTCTCTCGGTG | 5'TCACTGTTTCTAGCCTTC |
| RBBP9 | 5'ACGTTGGATG-CCTTTTTTTGGCTGGGTGTGACGTTG GAT GCCTTTTTT | 5'ACGTTGGATG-GCCTCAAGCAATACAACCACACG TTG GAT GGCCTC AA | 5'GGTGGCTCATGCCTGTA |
| CARD11 | 5'ACGTTGGATG-  AGATCACGCCACTGCACTC | 5'ACGTTGGATG-CCAGGTCAAGCTTGCCTTTT | 5'CAGCCTGGTGACAGAGC |
| MDM4 | 5'ACGTTGGATG-AAAAAAATCGTGGACCGGGC | 5'ACGTTGGATG-ACCTAGGTGATCTCCCAAAG | 5'AGTGGCTCACGCCTGTA |
| BRCA1 | 5'ACGTTGGATG-TATTTTGAGATGGAGTCCAGC | 5'ACGTTGGATG-GAAGTGCACGTTGCAGTGAG | 5'GTCGCCCAGGTTGGAGT |
| FANCC | 5'ACGTTGGATG-GGACTGGAGGAAGCACTAAT | 5'ACGTTGGATG-TGAGCCTCAGAGGTTGAGAC | 5'AGAGATGGGGTTTCACC |
